# Understanding the Mechanism of Bacterial Biofilms Resistance to Antimicrobial Agents

**DOI:** 10.2174/1874285801711010053

**Published:** 2017-04-28

**Authors:** Shriti Singh, Santosh Kumar Singh, Indrajit Chowdhury, Rajesh Singh

**Affiliations:** 1 Department of Kriya Sharir, Institute of Medical Sciences, Banaras Hindu University, Varanasi- 221 005 UP India; 2 Department of Microbiology, Biochemistry and Immunology, Morehouse School of Medicine, Atlanta, GA, USA; 3 Department of Obstetrics and Gynecology; Morehouse School of Medicine, Atlanta, GA, USA

**Keywords:** Biofilm, Quorum sensing, Efflux pump, Heterogeneity, Resistance

## Abstract

A biofilm is a group of microorganisms, that causes health problems for the patients with indwelling medical devices *via* attachment of cells to the surface matrix. It increases the resistance of a microorganism for antimicrobial agents and developed the human infection. Current strategies are removed or prevent the microbial colonies from the medical devices, which are attached to the surfaces. This will improve the clinical outcomes in favor of the patients suffering from serious infectious diseases. Moreover, the identification and inhibition of genes, which have the major role in biofilm formation, could be the effective approach for health care systems. In a current review article, we are highlighting the biofilm matrix and molecular mechanism of antimicrobial resistance in bacterial biofilms.

## 1 INTRODUCTION

Biofilm (a group of microorganism) having a complex assembly of protein, polysaccharide, and DNA in a self-produced extracellular polymeric matrix and was found on various surfaces, including, natural aquatic or potable water system, living tissues, medical devices, *etc.* [1]. Bacterial biofilms are widely studied in avoiding antibiotics, phagocytosis, and other disinfectant components. Biofilm communities in the natural environment have unique architectural features by interstitial voids, such as macro- and micro-colonies. The voids allowed the diffusion of nutrients, gasses, and antimicrobial agents through the biofilms; however, biofilm modulates their architecture in response to these changes in the external and internal process. Due to the proximity of cells, they exchange their quorum-sensing molecules, extra chromosomal plasmids and showed heterogeneous character in each biofilm community.

Microbial resistance acquired temporarily or the permanent ability of an organism when multiplying under circumstances and would abolish or inhibit other members of the same strain [1]. The antibiotics resistance is well known; however, food preservatives, disinfectants and resistance to antiseptics are comparatively under-explored. To regulate the gene expression in a coordinated fashion and mediate bacterial communication is also one of the hallmark mechanisms for resistance within a bacterial film [2]. The biofilm resistance of the microorganisms has several economic and environmental implications, including medical implants, oil recovery, drinking-water distribution, papermaking, metalworking and food-processing [3]. The bacterial attachments in the food and dairy industries are also well known related problems caused by biofilm mechanism [4]. Antimicrobial agents target a range of functional hereditary material, enzymes, respiratory system and other cellular loci. However, due to genetic exchanges and inherent discrepancies such as exclusive cell envelope composition and non-susceptible protein diverse bacteria, react differently to bactericides. Bacterial biofilm has increased antibiotic resistance and involved in many persistent diseases. Inside biofilm, several mechanisms confer the multi-factorial resistance to antibiotics. In this review, we are highlighting the molecular mechanism of biofilm and the role of the matrix for antimicrobial resistance.

## 2 RESISTANCE MECHANISMS OF BIOFILM

### 2.1 Capsules or Glycocalyx:

Glycocalyx is an integral part of the biofilms, and its thickness varied from 0.2 to 1.0μm [5] and was reported in both gram-positive and gram-negative bacteria [6]. Glycocalyx using electrostatic, Van Der Waal and hydrogen bonds forces for cohesion and adhesion of the biofilm with the solid surface [7] and that help in maturation of biofilm [8]. The composition of glycocalyx is flexible and regulated with biofilm growth that supports pathogenic bacteria to survive in extreme adverse host environment [9]. The components of the biofilm capsules such as glycoprotein and polysaccharides are influenced by in different environmental conditions. The resistance of bacteria against antibiotics and other components of antimicrobial agents are supported by glycocalyx matrix. Interestingly, glycocalyx layer accumulates antibacterial molecule up to 25% of its weight. The adsorption sites of the matrix limit the transportation of biocides and served an adherent for exoenzymes [10]. The exoenzymes protect the motility of particular agents of antibacterial activity and provide a source of substrate for biocide metabolite degradation that resulted slowing down the activity of susceptible drugs [11, 12].

### 2.2 Enzyme-Mediated Resistance

The transformation of bactericide to the nontoxic form is mediated by enzymes that provide resistance to biofilm. Few species of bacteria reported for degradation of the toxic compounds such as aromatic, phenolic and other heavy metals (nickel, cadmium, mercury, antimony, silver, copper, zinc, lead, cobalt, *etc.* [13]. Detoxification usually occurs by enzymatic reduction of ions and metal resistance genes. The presence of heavy metals induced the broader spectrum of resistant phenotype [14].

### 2.3 Heterogeneity in Metabolism and Growth Rate

The heterogeneities in an adapting population increase the chances that at least some individuals meet immediate or future challenges [15]. The growth rate and metabolic activity of the bacteria are affected by the differences in nutrients and oxygen availability within biofilms. The level of bacterial growth and activity inside biofilm was proved by different concentration of metabolic substrates and products [16]. Clostridia bacterium provides the valuable information for growth and fermentation in different cultivation condition [17]. This leads to heterogeneity of microbial population. The metabolic activities of cells were promoted by nutrients and oxygen in the periphery region of biofilm, which supports to bacteria in proliferation. In contrast, due to poor diffusion of nutrients, limits the metabolic potential inside niche resulted slowly growing the cells inside the biofilm matrix [18]. This was performed by the accumulation of guanine nucleotide-guanosine 3,5’-bis-pyro-phosphate (ppGpp) and the decreased level of RNA (tRNA and rRNA) synthesis. The concerning information on metabolic and growth rate heterogeneity of cells comes from the cellular enzyme synthesis within the biofilm [19]. The changes in the bacterial growth cycle influenced the level of enzyme synthesis in proportional to cell mass [20]. In stationary phase or slow growing bacteria cellular enzyme synthesis is arrested [21]. Biocides kill the metabolically active bacteria, whereas at the dormant growth phase, bacteria are less susceptible to the antimicrobial agents and protect them from the antimicrobial action [22]. However, in *E. coli* dormant growth phase synthesizing 1A- dependent ppGpp that suppresses the activity of autolysin and limits the cells anabolic process [23]. A mutation in *rel*A gene does not affect the growth rate, and such population was more sensitive to antibiotics. The population of *rel*A mutants inhibits peptidoglycan synthesis, which reduced the levels of activity of the bacteria cell wall inhibitors [24]. The increasing tolerance of bacterial biofilms towards antimicrobial agents reinforced the idea of metabolic growth rate heterogeneity. The metabolic activities are controlled by oxygen availability within biofilms. In *Pseudomonas aeruginosa,* bacterial biofilm is killed in pure oxygen when given ciprofloxacin and tobramycin antibiotics, however, reduction of oxygen availability enhanced the antibiotic resistance [25, 26]. Bacterial biofilm also increases the level of resistance against antibiotics through expressing specific genes under the anaerobic conditions.

### 2.4 Phenomenon of Persistence Shown by Cells

Persisters are the population of antimicrobial agent tolerant cells and are responsible for the severe chronic infectious disease. The detection of the bacterial strains are the major challenge in clinics. The study on ATP dependent persister formation conclude that lowering the ATP level decreases antibiotic target activity which leads to persister formation [27]. Bacterial biofilm contains resistant persister cells that exhibit multidrug and bactericidal agent tolerance [28]. Late growing gram positive or negative bacteria leading to tolerance or persistence, may exhibit to multidrug resistance and antibiotic tolerance.

Persisters cells formation controlled by the growth stages of bacterial communities, which are rapidly propagated and survive in the presence of lethal doses of antimicrobial agents [29]. Stationary phase bacteria produced a high level of persister cells and correlated with the increasing resistance inside biofilm [30]. The immune system eliminates the antibiotic action inside planktonic population, which is survived by persister cells [31]. Glycocalyx matrix helps the biofilm persisters to protect the immune system. After termination of the antibiotics in sessile bacterial population, persister cells start again re-inducing the growth of bacterial biofilm [32]. The stages of bacterial growth decided the formation of persisters [33]. One of the studies proves that persisters are in a loss if the stationary phase diluted [34]. The persisters’ formation also depends on the metabolic activity of bacteria [35] and suggested that persisters are a dormant variant of the wild type, not mutant cells. Interestingly, persisters not responded to bactericidal agent exposure [36]. Persisters compete for the antibiotic targets for the production of multidrug resistance (MDR) protein [36]. It is noteworthy that antibiotics act as a bactericidal with disrupting the function of target cells, rather than inhibition. Antibacterial compounds were leads to cell damage. The tolerance phenomenon of persisters has also been linked with programmed cell death (PCD) while the action of antimicrobial compounds leads the cell towards damage but not for complete cell death, which indirectly triggers PCD [35, 37]. Inside biofilm, autolysis is also the most common observation, which is performed by the peptidoglycan hydrolases called as autolysin [38]. Recently, Kirby-Bauer disk diffusion test was studied in late growing bacteria which detect the bacterial resistance and evaluate the level of tolerance by replacing the antibiotic disc impregnated with nutrients [39].

### 2.5 Metabolic State of the Organisms in the Biofilm

Biofilm resistance has been explained by the imposition of biofilm-specific growth within biofilm [40]. The physiological state of cells and the nature of the habitat can lead to considerable variation in the receptiveness of bacteria to bactericides. The limiting availability of nutrients affects the barrier composition and changes the bacterial cell envelope. After biofilm exposure to the inhibitory concentration of bactericides, resistant cell population leads to phenotypic adaptation. The heat or starvation stress in *E.coli* induces expression of resistance to UV light or H_2_O_2_. Another example of *Enterococcal* strains, after oxidative stress induction up regulate the expression of antioxidative enzymes and down regulate of prooxidative enzymes [41]. However, resistant phenotype becomes lost upon removal of bactericide. This has been suggested that a limitation of nutrient in biofilm causes to slow-growth and enter in the starved state [42]. The treatment with antimicrobial agents to biofilm leads to loss of their respiratory action due to their activity near the biofilm-bulk fluid interface. When the cells expanded as high growth rate rich media, the non-growing cells are less vulnerable to a variety of antimicrobial agents [43].

### 2.6 Genetic Adaptation

The genetic adaptation is required within biofilm to reduce susceptibility and to adopt the relatively protected and distinct phenotype. The multiple antibiotic resistance (*mar*) operons is a global regulator which controls the expression of various genes in *E. coli* and support multi-drug resistant phenotype includes antibiotics, organic solvents and the other disinfectants [44]. Moreover, *M. tuberculosis* adopts a dormancy state for decades in stressful environmental conditions and unrecognized by anti-TB drugs [45]. Most of the bacteria are fermentative, produces oxidant degrading and repairing enzymes and exhibit oxidizing stress response. These stress response cells become more resistant to a harmful factor within hours of exposure to sub-inhibitory quantities factor. Several defense genes have been characterized in *E. coli*, encoding catalysts; superoxide dismutases, hydroperoxide reductases, and alkyl glutathione reductases as well as DNA repair enzymes [46]. In addition, various regulatory genes such as*oxyR* and *soxR* have been characterized, which determine intracellular redox potential and activate a stress response when cells are subjected to oxidizing agents.

### 2.7 Quorum Sensing (Cell to Cell Signaling)

Quorum sensing (QS) is a process of the cell-to-cell interaction that regulates the behavior of bacteria. It depends upon extracellular signal molecules, detection, production, and autoinducers. Reboule *et al.* explore the family of molecules which belongs to natural compound that inhibited QS *via* antagonism of the receptor [47]. The bacteria by induction of a particular set of genes are capable of sensing and responding to increased cell population density [48]. Quorum sensing includes the production and secretion of an acyl homoserine lactones (AHL), which diffuse through the cell wall from the cell to the medium [49]. In gram-positive bacteria, quorum sensing secretes peptides as signal compounds and a two regulatory system (membrane-bound histidine kinase receptor and an intracellular response regulator) to detect the required changes in gene expression pattern and the peptides [48]. Besides, autoinducers-2 is another form of quorum sensing mechanism. These mechanisms are found in both gram-positive and negative bacteria [50]. The active cells in bacterial growth influenced by the glycocalyx matrix and degradative enzymes that regulate signaling molecule production such as S-adenosyl methionine and acyl-carrier proteins [51]. The mechanism of quorum sensing also reported in the control of biofilm maturation [52].

The role of signal molecule-mediated quorum sensing in biofilm formation has been demonstrated in many bacterial species. Quorum sensing systems influence the heterogeneous architecture of biofilm for the regulation of the degradative enzymes synthesis. Moreover, in suitable nutrient supply and environment, the expression of quorum sensing mediated phenotype is crucial in the cell migration and also protects from the deleterious environment of new modes of growth [53]. In Aeromonads, three QS regulate the expression of a function including biofilm formation, motility, and virulence and harbor the differences in other influences [47]. Signaling mechanism from cell-to-cell in *Pseudomonas aeruginosa*, controls the expression of superoxide dismutase and catalase genes, which mediate the resistance to hydrogen peroxides [53]. The quorum sense deficiency is correlated with thinner biofilm formation and lower EPS production, and such a mutant or deficient biofilm is susceptible to kanamycin [54]. These studies suggested that quorum-sensing system responded to biofilm either directly or indirectly to environmental stress [55]. Another study of *Pseudomonas aeruginosa* shown, that it kills the other competing bacteria through the 2-heptyl-3-hydroxy-4-quinolone signal using stored bacterial iron. The previous study on *Pseudomonas* quinolone has also shown higher affinity signal with iron chelator. Schertzer *et al.* found the similarity in action between glycocalyx matrix and signal molecules to trap external positive-charged compounds [56].

### 2.8 Stress Responses

A stress response in biofilm, characterized by numerous changes in bacterial physiology and morphology that increase the cellular stress resistance [57]. In general, stress response controls the formation of cell envelope and synthesis of thin aggregative fimbriae in *E. coli* and *Salmonella enteritis*ser ovar *Typhimurium*. In addition, the stress response functions as a preventive factor for cellular damage rather than repair. Several factors were found responsible for stress induction such as nutrient deprivation caused by stationary phase bacteria growth, high or low temperature, higher osmolality and acidic pH [58-60]. Interestingly, RpoS, a sigma subunit of RNA polymerase is found to induce in *E. coli* when exposed to adverse environmental conditions. The same sigma factor is found to control 50 genes that determine stress tolerance to cells while others regulate the physiological rearrangement or redirect the metabolism of bacteria upon stress condition. The alteration of gene expresses due to the general stress response in the cells immobilized in the biofilm matrix, may result in increasing resistance to biocides action [61]

### 2.9 Outer Membrane Structure

As antibacterial agents have to penetrate to reach the target site; modification of cell envelope or adaptation is found to be responsible for bacterial cell resistance to antibiotics. Hydrophilic antibacterial agents are mainly prevented from entering through the outer membrane by the lipopolysaccharide layer and the underlying phospholipids, whereas outer membrane proteins exclude hydrophobic agents. Moreover, Gram-positive bacteria (*Nocardia farcinica)* has a complex cell wall and bind to a variety of lipids and pore-forming proteins and form a hydrophilic pathway across the cell wall and exhibited in interaction with the antibiotics. The role of mycobacterial resistance to antibiotics in outer-membrane is indispensable [62]. Certain antibiotic-resistant bacteria strain either lack or over express outer membrane proteins. For example, *P. aeroginosa* strain lacks Opr D, a porin selective for certain carbon sources [63], which are the port of entry for isothiazolone. Alteration in the outer membrane protein profile leads to the exclusion of bactericide sodium dimethyl dithiocarbonate (SMT).

### 2.10 Efflux Pumps

A set of efflux systems facilitates bacterial survival under extreme conditions, including antimicrobial agents. Efflux pumps exert both intrinsic and acquired resistance to different antibacterial agents that belong to same or different families [64, 65]. Overproduction of efflux pump can lead to multidrug resistance. Bacterial efflux pumps exert multidrug resistance (MDR) phenotype combined with other resistance mechanisms such as target modification and antibiotic inactivation [66, 67]. *Acinetobacter baumannii* is a major challenge in the hospital, which is reported in multidrug resistance, that persists in the environment, and forms a biofilm on wound surface [68]. Besides, gram-positive bacteria (*Bacillus subtitles, L. lactic, S. aureus* and *etc*.) also causes severe human infections due to resistance behavior towards antimicrobial agents [69]. These possess multiple drug resistance mechanisms and transport multiple unrelated compounds that result a multidrug resistance (MDR) phenotype. In several pathogenic bacteria such as *Escherichia coli (E. coli)*, *Enterobacteraerogenes* and *Klebsiella pneumonia,* the efflux pump slows down the penetration of hydrophilic solutes that decrease the transmembrane diffusion of lipophilic solutes by down-regulating the ‘porin’ production [70, 71]. Several studies have reported the localization of the efflux pump-encoding genes on either chromosome of plasmids exerting resistance to various antibiotics as well as biocides, dyes, and detergents [72].

Five different classes of bacterial efflux pumps have been identified such as the major facilitator superfamily (MF), the resistance-nodulation-division family (RND), the small multidrug resistance family (SMR), the ATP-binding cassette family (ABC) and the multidrug and toxic compound extrusion family (MATE) [73]. To drive antimicrobial agent efflux, the ABC family system hydrolyses ATP, whereas the MF family, MATE family, and the RND family functions as secondary transporters, catalyzing drug ion antiproton (H+ or Na+) [74]. RND family transporters are the first line of defense in bacteria by serving the target mutation or drug modification.

Exposure of the bacterial biofilm to lower concentrations of antibiotics, such as chloramphenicol and tetracycline and to xenobiotic such as salicylate and chlorinated phenols, induces the expression of multi-drug resistance operons and efflux pumps [75, 76]. DNA microarray analysis of mature *Pseudomonas aeruginosa* PA01 biofilm demonstrated that none of the genes encoding the RND efflux system when induced in sessile bacterial population grown in antibiotic-free environments [77, 78]. Similarly, multidrug resistance phenotype in *E. coli* biofilm is regulated by *mar* and *acr*AB encoding genes. Several antibiotics such as penicillin’s, cephalosporin’s, rifampicin, nalidixic acid and fluoroquinolones and oxidative stress agents up regulate *mar* in planktonic bacteria exerting a resistance phenotype [79]. In addition, sub-lethal doses of several commonly used medicinally important antibiotics such as tetracycline; chloramphenicol, salicylate and paracetamol can induce *mar* expression level [80, 81].

The *acr*AB efflux pump determined the multidrug resistant phenotype of *mar* mutant isolates and also reported as up regulated in *mar* mutants [82]. Lower susceptibility of *E. coli* biofilm to sub-lethal doses of ciprofloxacin is enhanced through constitutive expressions of acrAB efflux pump. In addition, the stationary phase of bacterial growth is related to the expression of *mar* and its target genes. It was observed that metabolic activity of bacteria was highly suppressed with the higher expression level of *mar* in *E. coli* biofilm. Mutants of *Pseudomonas aeruginosa* with increased expression of efflux pumps play a significant role in decreasing susceptibility of *Pseudomonas aeruginosa* biofilm to antibiotics [83]. The Efflux pumps are the major hurdles in drug discovery and the main player in multidrug resistance of gram-negative bacteria. The recent advances in the understanding of efflux pumps could provide the drug discovery platform in bacteria [84].

## CONCLUSION AND FUTURE PROSPECTS

**Fig. (1):**
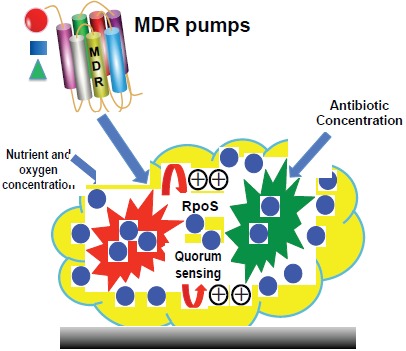
Mechanisms of resistance in bacterial biofilm.

Several factors contribute to being the development of resistance to bacteria in biofilms and varied among microorganisms (Fig. **1**). Antibiotics have been widely used at the variable concentration in worldwide that may affect the microbiota, consequences of that alteration of genes, mutation, drug resistance and also human health [85]. However, Biofilm is more protective and resistance to the action of antibiotics. It is important to remark that surface associated infections (prosthesis, catheters, lung, *etc.*) increased by bacterial colonization and physiological alteration inside biofilm. The device related infections are major hurdles in the treatment of the patient that expose to high tolerance antibiotics. The bacteria originated from biofilm can spread into bloodstream or other infection. Thus, bacteria embedded in a biofilm are able to withstand to antibiobiotics and influence the patient management. Moreover, Gram-positive and Gram-negative bacteria form highly tolerant persisters in the biofilms, which inferred the high risk of that infection recurrence during biofilm infections [86]. Intensive research on the regulation of gene expression is currently ongoing that provides recognition and transfer of multidrug efflux proteins.

The increased antibiotic resistance of biofilm is due to (i) limited diffusion of antimicrobial agents through the biofilm matrix, (ii) communication of the antimicrobial agents with the biofilm matrix (polymer and cells), (iii) enzyme-mediated resistance, (iv) levels of metabolic activity inside the biofilm, (v) genetic adaptation, (vi) efflux pumps and (vii) outer membrane structure. The antibiotic resistance is supported due to the transition of the colony from exponential to slow or without growth/persisters phenomena. The Glycocalyx matrix through the efflux system and enzymes, inactivate antimicrobial agents and protect the peripheral region of the biofilm. Interestingly, cells in the intermediate position of a biofilm, starve for a particular nutrient and slow their growth. The changes at various gene expression levels due to stress response to severe conditions prevent the surface-bound bacteria from cellular damage. A detailed mechanism of biofilm resistance can be investigated by isolating the antibiotic-resistant bacteria occurring in the biofilms. Also, new investigations will improve patient health and survival, the complex interaction between the biofilm communities and the host defense system.
